# Identification of N6-Methyladenosine-Associated Long Non-coding RNAs for Immunotherapeutic Response and Prognosis in Patients With Pancreatic Cancer

**DOI:** 10.3389/fcell.2021.748442

**Published:** 2021-09-21

**Authors:** Xinshuang Yu, Peng Dong, Yu Yan, Fengjun Liu, Hui Wang, Yajuan Lv, Meijuan Song, Qingqiang Yao, Sanyuan Hu

**Affiliations:** ^1^Department of Oncology, The First Affiliated Hospital of Shandong First Medical University & Shandong Provincial Qianfoshan Hospital, Shandong Key Laboratory of Rheumatic Disease and Translational Medicine, Shandong Lung Cancer Institute, Jinan, China; ^2^School of Medicine, Cheeloo College of Medicine, Shandong University, Jinan, China; ^3^Institute of Materia Medica, Shandong First Medical University & Shandong Academy of Medical Sciences, Jinan, China; ^4^Shandong First Medical University & Shandong Academy of Medical Sciences, Jinan, China; ^5^Department of General Surgery, The First Affiliated Hospital of Shandong First Medical University & Shandong Provincial Qianfoshan Hospital, Key Laboratory of Metabolism and Gastrointestinal Tumor, The First Affiliated Hospital of Shandong First Medical University, Key Laboratory of Laparoscopic Technology, The First Affiliated Hospital of Shandong First Medical University, Shandong Medicine and Health Key Laboratory of General Surgery, Jinan, China

**Keywords:** N6-methyladenosine (m6A), long non-coding RNA (lncRNA), pancreatic cancer, immunogenomic landscape, survival

## Abstract

Pancreatic cancer is a highly aggressive disease with poor prognosis. N6-methyladenosine (m6A) is critical for post-transcriptional modification of messenger RNA (mRNA) and long non-coding RNA (lncRNA). However, the m6A-associated lncRNAs (m6A-lncRNA) and their values in predicting clinical outcomes and immune microenvironmental status in pancreatic cancer patients remain largely unexplored. This study aimed to evaluate the importance of m6A-lncRNA and established a m6A-lncRNA signature for predicting immunotherapeutic response and prognosis of pancreatic cancer. The m6A-lncRNA co-expression networks were constructed using data from the TCGA and GTEx database. Based on the least absolute shrinkage and selection operator (LASSO) analysis, we constructed an 8 m6A-lncRNA signature risk model, and selection operator (LASSO) analysis, and stratified patients into the high- and low-risk groups with significant difference in overall survival (OS) (HR = 2.68, 95% CI = 1.74–4.14, *P* < 0.0001). Patients in the high-risk group showed significantly reduced OS compared to patients in the low-risk group (*P* < 0.001). The clinical characteristics and m6A-lncRNA risk scores were used to construct a nomogram which accurately predicted the OS in pancreatic cancer. TIMER 2.0 were used to investigate tumor immune infiltrating cells and its relationship with pancreatic cancer. CIBERSORT analysis revealed increased higher infiltration proportions of M0 and M2 macrophages, and lower infiltration of naive B cell, CD8^+^ T cell and Treg cells in the high-risk group. Compared to the low-risk group, functional annotation using ssGSEA showed that T cell infiltration and the differential immune-related check-point genes are expressed at low level in the high-risk group (*P* < 0.05). In summary, our study constructed a novel m6A-associated lncRNAs signature to predict immunotherapeutic responses and provided a novel nomogram for the prognosis prediction of pancreatic cancer.

## Introduction

Pancreatic cancer is one of the most life-threatening malignant cancers. It is difficult to be diagnosed in the early stage and is progressed rapidly by about 1% per year (Maisonneuve, 2019; Romano et al., 2021; Siegel et al., 2021). In 2020, there were a total of 496,000 new cases and 466,000 deaths (Sung et al., 2021). New cases arise to 60,430, and cancer related death arise to 48,200 in 2021 (Romano et al., 2021). The median survival time of pancreatic cancer is 4–5 months and the 5-year survival rate for pancreatic cancer remains at 8.2% (Geng et al., 2020). Symptoms of early stage pancreatic cancer are often indistinct and difficult to be identified. Therefore, it is crucial to find out new diagnostic indicators for early detection and treatment of pancreatic cancer.

N6-methyladenosine (m6A) was first reported in the 1970s (Desrosiers et al., 1974). The m6A mediated greater than 60% RNA methylation in post-transcriptional modification of mRNA in eukaryotes (Yang et al., 2018; Du et al., 2019; Lv et al., 2020). Abnormal m6A methylation occupies a prominent position in both normal biological and cellular regulation and tumorigenesis (Chen et al., 2018). Following the development of MeRIP-seq (methylated RNA immunoprecipitation sequencing) and miCLIP (m6A individual nucleotide-resolution cross-linking and immunoprecipitation) technologies, lncRNAs (long non-coding RNA) are found to participate in the regulation of m6A modification (Fazi and Fatica, 2019).

LncRNAs belong to non-coding RNAs and are longer than 200 nucleotides in length. LncRNA interact with m6A (Lv et al., 2017; Dai et al., 2020), and crosstalk between m6A modifications and lncRNAs (comprise the majority of ncRNAs) contribute to tumorigenesis (Fazi and Fatica, 2019). Moreover, recent studies (Denaro et al., 2019; Sun et al., 2020) have demonstrated that lncRNAs are critical in the regulation of cancer immunity and in the development and differentiation of different immune cell lineages. m6A associated lncRNA-based signatures involved in prognostic prediction and immune regulation has become a focus of research, but there are few reports about the relationship between m6A-lncRNA and the immune status of the tumor microenvironment in pancreatic cancer.

In the present study, we aimed to construct an m6A-related lncRNA (m6A-lncRNA) signature and nomogram using bioinformatics approach to identify its relationship with immune cell and immune microenvironment, and to explore the potential of using m6A- lncRNA as predictive biomarkers for prognosis and immunotherapy in pancreatic cancer.

## Materials and Methods

### Acquisition of Information of Pancreatic Cancer Data From Public Database

A portion of pancreatic cancer samples and the corresponding clinical data were obtained from the TCGA PAAD database^1^ and the data of normal was obtained from GTEx database.^2^ A cohort of 178 pancreatic cancer and 171 normal pancreatic tissue from TCGA and GTEx were included for analysis. Samples with missing OS values or with OS ≤ 30 days were excluded (Subramanian et al., 2005). In total, 177 pancreatic cancer patients samples defined as a combination set, which was divided into a training set and a validation set.

### Identification of N6-Methyladenosine-Associated Long Non-coding RNAs and Novel Candidate of Pancreatic Cancer

Twenty-five m6A RNA methylation were collected from the m6A2target database^3^ (Deng et al., 2021) and literatures (Tu et al., 2020; Xu et al., 2020). The expression matrixes of these 25 m6A genes were retrieved from the TCGA, including the expression regulatory factors of writers (METTL3, METTL14, METTL16, CBLL1, ZCCHC4, WTAP, VIRMA, ZC3H13, RBM15, and RBM15B), readers (YTHDC1, YTHDC2, YTHDF1, YTHDF2, YTHDF3, HNRNPC, FMR1, RBMX, HNRNPA2B1, LRRPRC, IGF2BP1, IGF2BP2, and IGF2BP3), and erasers (FTO and ALKBH5). Then, a cohort of m6A-related lncRNAs was identified according to Pearson correlation analysis between the m6A genes and lncRNA expression level in samples (| R| > 0.5, *P* < 0.01).

### Establishing of the N6-Methyladenosine-Associated Long Non-coding RNAs Risk Signature

The TCGA pancreatic cancer data set was randomized as a training set and a validation set. The training set was to construct an m6A-associated lncRNA model, and the validation set was applied to validate this established model. Univariate Cox regression analysis was performed to sort the m6A-associated lncRNAs with significant prognostic value (*P* < 0.01). The low- and high-risk groups were divided using the median risk score. Multivariate Cox regression analysis was used to evaluate the independent prognostic factors in pancreatic cancer. Then, the prognosis-related gene sets from m6A-associated lncRNAs were further analyzed by the least absolute shrinkage and selection operator (LASSO) regression analysis. The m6A-lncRNA significantly associated with prognosis were obtained, and the risk characteristics of each sample were constructed. The samples were split into two groups: a high- and a low-risk group according to the characteristics and coefficients, Receiver operating characteristic (ROC) curve was generated to analyze the 1 year survival rate of patients and assess the accuracy of survival prediction of the gene signature.

### Construction and Evaluation of a Predictive Nomogram

The predictive ability of the nomogram and other predictors (age, gender, grade, clinical stage, T status, N status, Alcohol take, Radiation, Chemotherapy, and risk score) for the 1-, 2-, and 3-year OS was set up. The ROC curve and C-index (Harrell’ concordance index) was used to evaluate the best prediction of the model.

### Estimation Clinical Feature and Tumor Immune Microenvironment Profile Using the N6-Methyladenosine-Associated Long Non-coding RNAs Model

To evaluate the biological characteristics of the m6A-lncRNAs in pancreatic cancer, the relationship between high- and long-risk groups and clinical features was further examined. The candidate m6A-lncRNA gene sets were presented as feature factors Clinicopathological characteristics (age, gender, grade, TNM staging, T status, N status, alcohol take, diabetes history, radiation, chemotherapy) between groups were compared in the TCGA set.

To evaluate the immune cell infiltration data and its related immune function in pancreatic cancer, we download infiltrating immune cell data from TIMER 2.0^4^ (Li et al., 2017). CIBERSORT (Newman et al., 2015) algorithm was also applied to calculate the infiltrating ratio of 22 immune cell types in tumor samples. The activity of 13 immune-related pathways were calculated with ssGSEA (Li et al., 2017; Yi et al., 2020). The potential immune check-point molecules were retrieved from published literature (Tang et al., 2021).

## Results

### Characteristics of N6-Methyladenosine-Associated Long Non-coding RNAs in Pancreatic Cancer

The study flow chart is shown in Supplementary Figure 1. The detailed clinical characteristics of these patients are summarized in Table 1. A total of 25 m6A methylation regulators were divided into three types: binding protein (readers); methyltransferase (writers); and demethylase (erasers) according to their roles in the methylation process. The 25 m6A RNA methylation were listed in Table 2.

**TABLE 1:** Clinical characteristics of the pancreatic cancer patients.

Characteristics	Type	Total	Test	Train	*P*-value
**Age**	≤65	93(52.54%)	52(59.09%)	41(46.07%)	0.1131
	>65	84(47.46%)	36(40.91%)	48(53.93%)	
**Gender**	Male	97(54.8%)	50(56.82%)	47(52.81%)	0.7004
	Female	80(45.2%)	38(43.18%)	42(47.19%)	
**Grade**	G1–2	125(70.62%)	66(75%)	59(66.29%)	0.4347
	G3–4	50(28.25%)	21(23.86%)	29(32.58%)	
	Unknown	2(1.13%)	1(1.14%)	1(1.12%)	
**T**	T1–2	31(17.51%)	10(11.36%)	21(23.6%)	0.1007
	T3–4	144(81.36%)	77(87.5%)	67(75.28%)	
	Unknown	2(1.13%)	1(1.14%)	1(1.12%)	
**N**	N0	49(27.68%)	23(26.14%)	26(29.21%)	0.8244
	N1	123(69.49%)	62(70.45%)	61(68.54%)	
	Unknown	5(2.82%)	3(3.41%)	2(2.25%)	
**Stage**	Stage I–II	167(94.35%)	84(95.45%)	83(93.26%)	0.788
	Stage III–IV	7(3.95%)	3(3.41%)	4(4.49%)	
	Unknown	3(1.69%)	1(1.14%)	2(2.25%)	
**Alcohol**	Yes	101(57.06%)	51(57.95%)	50(56.18%)	0.8447
	No	64(36.16%)	32(36.36%)	32(35.96%)	
	Unknown	12(6.78%)	5(5.68%)	7(7.87%)	
**Diabetes**	Yes	38(21.47%)	21(23.86%)	17(19.1%)	0.6767
	No	108(61.02%)	51(57.95%)	57(64.04%)	
	Unknown	31(17.51%)	16(18.18%)	15(16.85%)	
**Radiation**	Yes	32(18.08%)	18(20.45%)	14(15.73%)	0.6901
	No	101(57.06%)	48(54.55%)	53(59.55%)	
	Unknown	44(24.86%)	22(25%)	22(24.72%)	
**Chemotherapy**	Yes	109(61.58%)	55(62.5%)	54(60.67%)	0.6287
	No	62(35.03%)	29(32.95%)	33(37.08%)	
	Unknown	6(3.39%)	4(4.55%)	2(2.25%)	

**TABLE 2:** The components of m6A RNA methylation regulators in writer-, reader,- and eraser-complex.

Regulators	Gene set
Writers(10)	METTL3, METTL14, METTL16, CBLL1(HAKAI), ZCCHC4, ZC3H13, VIRMA (KIAA1429), WTAP, RBM15, and RBM15B
Readers(13)	HNRNPC, YTHDF1, YTHDF2, YTHDF3, YTHDC1, YTHDC2, FMR1 (FMRP), RBMX (HNRNPG), HNRNPA2B1, LRPPRC, IGF2BP1, IGF2BP2, IGF2BP3
Erasers(2)	FTO, ALKBH5

**FIGURE 1:**
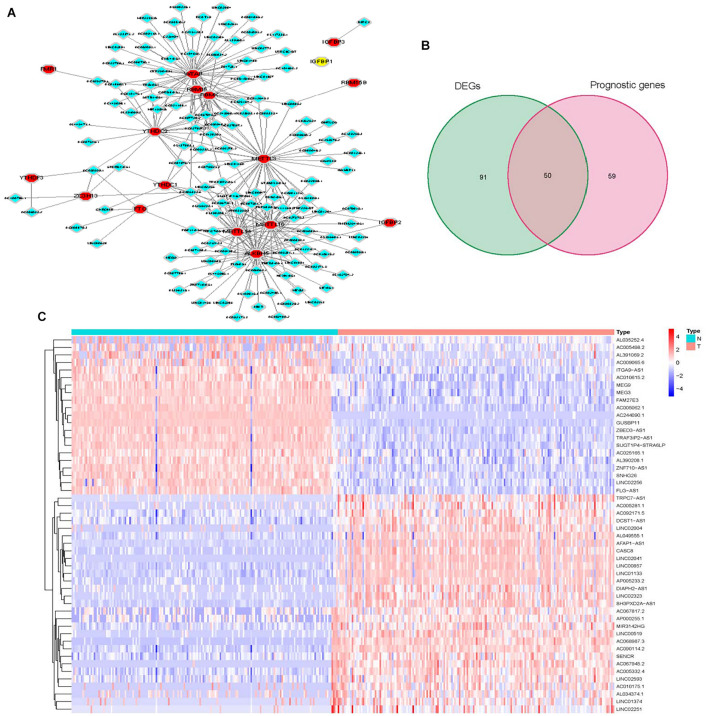
Identification of m6A-associated lncRNAs in pancreatic cancer. **(A)** Co-expression network of m6A regulatory genes and related lncRNA. **(B)** Venn plot of differential and prognostic m6A-associatedlncRNAs. **(C)** Heatmap for prognosis related differential m6A-lncRNAs (hub genes).

To identify m6A-associated lncRNAs in pancreatic cancer, we use co-expressed perl script to identify 276 m6A-associated lncRNAs by constructing m6A-lncRNA co-expression networks based on the available TCGA dataset. The overall features of m6A-associated lncRNAs were shown in Figure 1A. Among them, 51% (141/276) of the m6A-associated lncRNAs were differentially expressed between tumor tissues and adjacent non-tumorous tissues, and the univariate Cox regression analysis showed that 39.4% (109/276) of the m6A-associated lncRNAs were correlated with OS Then we extracted these prognosis related differential m6A-lncRNAs (hub genes) for further analysis (Figures 1B,C).

### Risk Model of N6-Methyladenosine-Long Non-coding RNA Signature and Patients’ Survival

**FIGURE 2:**
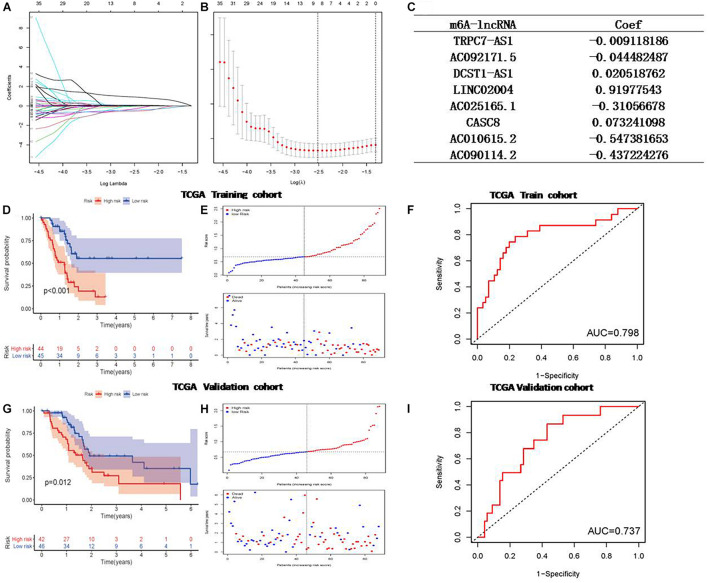
Least absolute shrinkage and selection operator (LASSO) regression for calculating risk model for pancreatic cancer patients based on the m6A-associated lncRNAs. **(A)** The tuning parameters of OS-related proteins to cross-verify the error curve. **(B)** Perpendicular imaginary lines to calculating the minimum criteria **(C)** The LASSO coefficient profile of 24 OS-related m6A-lncRNA and perpendicular imaginary line. **(D)** Survival status of patients in different groups in training cohort. **(E)** Rank of prognostic index and distribution of groups based on m6A-lncRNA prognostic signature risk scores in training cohort. **(F)** Receiver operating characteristic (ROC) curves of the m6A-lncRNA prognostic signature for predicting the 1-year survival in training cohort. **(G)** Survival status in validation cohort. **(H)** Rank of prognostic index and distribution of groups based on m6A-lncRNA prognostic signature risk scores in validation cohort. **(I)** ROC curves for predicting the 1-year survival in validation cohort.

LASSO Cox regression analysis was used to construct a prognostic model based on the expression profile of the prognostic related m6A-lncRNA genes mentioned above. As a result, 8 of the prognostic related m6A-lncRNA genes were significantly correlated with OS with *p* < 0.05, including TRPC7-AS1, AC092171.5, DCST1-AS1, LINC02004, AC025165.1, CASC8, AC010615.2, and AC090114.2. Then the LASSO Cox regression analysis for those 8 m6A-lncRNAs was performed to establish a comprehensive risk signature for prognosis. The weighed summation of gene expression levels of constituent biomarkers, i.e., the risk score, for tumor samples were calculated based on the coefficients determined by the LASSO Cox regression analysis (Figure 2C). The tuning parameters (log λ) of associated molecules were selected to cross-verify the error curves. According to the minimal criterion and 1-se criterion, perpendicular imaginary lines were drawn at the optimal value (Figure 2A). The LASSO coefficient profile of 109 OS-related lncRNAs and perpendicular imaginary line were drawn at the value chosen by 10-fold cross-validation (Figure 2B). Finally, the pancreatic cancer patients data from TCGA database were divided into a high-and low-risk score groups for further assessment.

**FIGURE 3:**
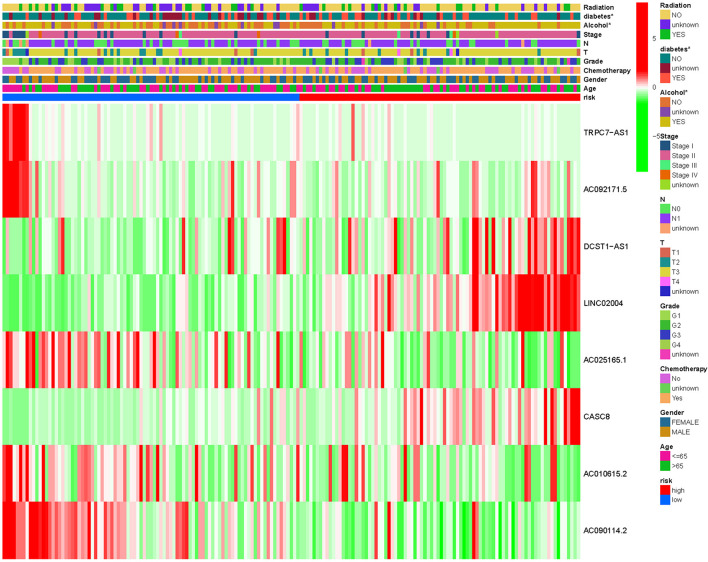
Heatmap for the m6A-associated lncRNAsprognostic signature and clinicopathological manifestations in pancreatic cancer. *P < 0.05.

Kaplan-Meier analysis was performed to evaluate. The prognostic value of the 8m6A-lncRNA signature in these pancreatic cancer patients was evaluated by the Kaplan-Meier analysis. To test the robustness of the model constructed from the TCGA validating cohort, the patients were categorized into high- or low-risk groups by the median value calculated with the same formula as that from the TCGA training cohort. The risk plot and the survival status of patients between the low-risk and high-risk groups were distributed in discrete directions both in the training set (Figure 2E) and in the validation set (Figure 2H). High m6A-lncRNA risk score was associated with poor OS in the TCGA training set (Figure 2D), and this result was further validated by the validation set (Figure 2G). Finally, a 8 m6A-lncRNA gene signature was constructed to stratify patients into two risk groups with significantly different OS (HR = 2.68, 95% CI = 1.74–4.14, *P* < 0.0001). One year ROC curve was performed, and it showed good sensitivity and specificity of survival prediction both in the training group (Figure 2F) and validation group (Figure 2I). The clinicopathological features between the two were compared and shown in Figure 3, and it indicates that diabetes history and alcohol uptake associated with pancreatic risk.

### Construction and Evaluation of the Prognostic Nomogram

**FIGURE 4:**
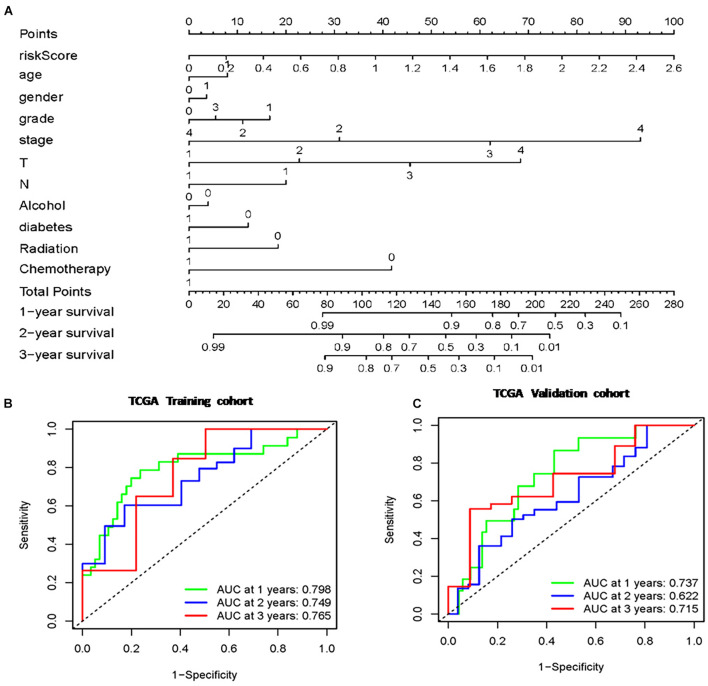
Nomogram based on risk score of m6A-lncRNA signature and clinicopathological manifestation. **(A)** The nomogram predicts the probability of the 1-, 2-, and 3-year OS. **(B)** The ROC of the TCGA training cohort **(C)** The ROC of the TCGA validation cohort.

A total of 177 samples were included in the analysis after deleting those with no proper follow-up. These TCGA samples included 80 females and 97 males. Also the samples included datas 93 were under 65-year-old and 84 were older than 65-year-old. A brief summary of clinical and pathological characteristics was shown in Table 1. The clinical characteristics and m6A-lncRNA risk score were used to construct a prognostic nomogram (Figure 4A). Multivariate Cox regression analyses were carried out among the available variables to determine whether the 8 m6A-lncRNA risk score was an independent prognostic predictor for OS (training: HR = 4.287 95% CI = 1.903–9.658, *P* < 0.001; validation: HR = 2.915, 95% CI = 1.096–7.755, *P* = 0.03; Table 3). Each factor (age, gender, grade, clinical stage, T status, N status, Alcohol take, Diabetes history, Radiation, Chemotherapy, and m6A-lncRNA risk score) was used to obtain a summary score and the total score of the individual sample. The 8 m6A-lncRNA risk score and the clinicopathologic features were used to predict the 1-, 2-, and 3- year OS rates of pancreatic cancer patients. The true positive vs. false positive of the prediction with AUC reached 0.798 at 1 year, 0.749 at 2 years, and 0.765 at 3 years in the training group (Figure 4B). The AUC reached 0.737 at 1 year, 0.622 at 2 years, and 0.715 at 3 years in validation group (Figure 4C), suggesting good prediction performance. The C-index used to evaluate the model was 0.80, indicating that the nomogram has a good fit.

**TABLE 3:** Univariate and multivariate regression analysis of pancreatic cancer patients in each data set.

Univariate analysis				Multivariate analysis			Univariate analysis			Multivariate analysis		

Variables	HR	95%CI of HR	*P*-value	HR	95%CI of HR	*P*-value	HR	95%CI of HR	*P*-value	HR	95%CI of HR	*P*-value
**Training set**							**Validation set**					
Age	1.03	0.995–1.058	0.095	0.99	0.957–1.020	0.459	1.03	0.986–1.074	0.193	1.05	1.004–1.092	0.031
Gender	1.01	0.502–2.035	0.975	1.46	0.670–3.198	0.339	0.88	0.427–1.771	0.7	0.87	0.314–2.427	0.795
Grade	1.73	1.041–2.879	0.034	1.22	0.671–2.234	0.51	1.11	0.684–1.805	0.671	1	0.515–1.944	0.998
Stage	4.38	1.035–18.552	0.045	2.24	0.474–10.602	0.308	2.05	0.998–4.224	0.051	2.24	1.005–4.993	0.049
Smoke	1.38	0.186–10.175	0.751	0.55	0.064–4.822	0.592	1.21	0.284–5.129	0.798	1.91	0.287–12.671	0.505
Alcohol	1.32	0.638–2.749	0.451	0.92	0.391–2.171	0.852	1.19	0.513–2.775	0.682	1.03	0.326–3.239	<0.001
Diabetes	1.04	0.448–2.402	0.933	1.82	0.721–4.591	0.205	0.86	0.367–2.001	0.72	0.39	0.141–1.071	0.068
Radiation	0.15	0.036–0.627	0.009	0.21	0.040–1.092	0.063	0.67	0.289–1.570	0.36	0.29	0.100–0.845	0.023
Risk score	4.3	2.410–7.684	<0.001	4.29	1.902–9.658	<0.001	2.73	1.170–6.387	0.02	2.92	1.096–7.755	0.032

### Evaluation of Tumor Infiltrating Lymphocytes Landerscape

**FIGURE 5:**
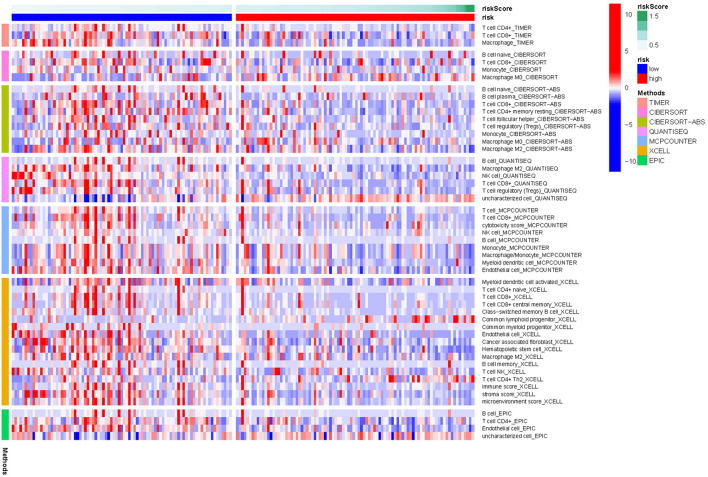
Heatmap for immune responses based on TIMER 2.0 algorithms in pancreatic cancer between the high- and low- risk groups.

The cellular components and immune responses between the high-and low-risk groups were assessed. TIMER 2.0 to calculate the tumor infiltrating cells, and single-sample gene set enrichment analysis (ssGSEA) were also used to quantify immune infiltration based on the M6A-lncRNA signature. The differences in immune response under different algorithms was shown using a heatmap in Figure 5.

**FIGURE 6:**
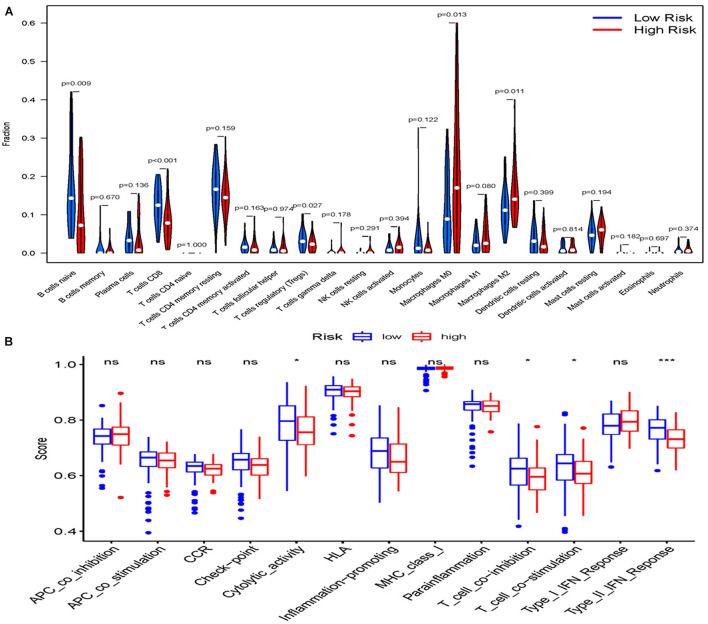
Landscape of Immune Cell Infiltration in pancreatic cancer. **(A)** The infiltration of 22 immune cell types in the high- and low-risk groups in the TCGAcohort. **(B)** Comparison of the 13 immune-related functions between the high-and low-risk groups in the TCGA cohort. Adjusted *P*-values were showed as: ns, not significant; *P < 0.05; ***P < 0.001.

The infiltrating score of 22 immune cells were displayed in Figure 6A. It was found that patients who had high m6A-lncRNA signature risk had significantly higher infiltration proportions of M0 and M2 macrophages, and lower infiltration of naive B cell CD8^+^ T cell and Treg cells. Correlation analysis between immune cell subpopulations and related functions based on ssGSEA revealed that T cell functions (including Cytolytic activity, T cell co-inhibition and co-stimulation, and type II IFN response) were significantly lower in the high- risk group than in the low-risk group (all adjusted *P* < 0.05, Figure 6B). These results indicate that high m6a-lncRNA risk group is accompanied with an immune-deficient status, especially for the T cell function.

**FIGURE 7:**
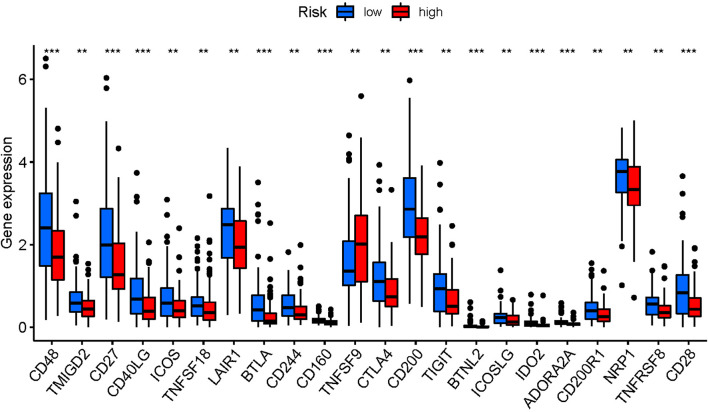
Expression of immune checkpoints among high- and low-risk pancreatic cancer groups. *P*-values were showed as: ns, not significant; **P < 0.01; ***P < 0.001.

The difference in the expression of immune checkpoints genes between the two groups was also explored, due to the importance of checkpoint inhibitor-based immunotherapies. We found that the immune-related genes were low in the high risk group, indicating that immune checkpoint inhibitors may not be sensitive in pancreatic cancer patients with immunotherapy (Figure 7).

## Discussion

Pancreatic cancer is an extremely heterogeneous and universally fatal disease and has been reported to be one of the most common causes of cancer-related death worldwide (Lanfranca et al., 2019; Siegel et al., 2021). Despite the progress made in surgery, radiotherapy and chemotherapy, the prognosis of advanced pancreatic cancer is still poor. Pancreatic cancer is very difficult to detect in the early stage. Therefore, there are increasing interests to explore reliable prognostic biomarkers to better identify patients with high risk of the disease, who would benefit from intensive treatment. In this study, we integrate transcriptome data and corresponding clinical data on 25 m6A methylation-related genes and m6A-related lncRNAs from the TCGA and the GTEx database. We systematically investigated the expression ofm6A-lncRNA in pancreatic tumor tissues and associations with OS. A prognostic model of 8 m6A-lncRNAs was constructed, and this model was validated in an internal cohort.

The m6A methylation is important in common mRNA modification and cancer pathogenesis. m6A modification of lncRNAs can affect the occurrence, development and progression of tumors (Zhou et al., 2016). Similar to mRNAs, lncRNAs are modulated by m6A and exert their regulatory roles by affecting the expression of protein-coding genes (Han et al., 2019; Xiao et al., 2019). Thus, identifying m6A-associated genes and m6A RNA methylation regulators, particularlym6A-associated lncRNA, in deadly pancreatic cancer may provide valuable therapeutic targets.

In the present study, we identified key genes from 276 candidate m6A related lncRNAs and explored their significance in the clinical features of pancreatic cancer patients. We established the m6A-lncRNA model. This model was able to evaluate the prognosis of patients with pancreatic cancer, which is of great significance for the clinical diagnosis and treatment of patients. Nomograms are widely used in the evaluation of tumor prognosis. The degree to which the various factors in the model contribute to the prognostic outcome is scored, and the prognostic outcome events are calculated by determining the total score of the different factors. The main advantage of a nomogram is that it individualized risk assessments according to the characteristics of patients or diseases. In this study, we constructed a prognostic nomogram combining clinical features with m6A-lncRNA signature.

Pancreatic cancer is a disease associated with immunosuppression. Currently, little is known about the m6A -lncRNA on TIME in pancreatic cancer is limited. A previous study indicated that immune infiltrating cells can be modulated by the immune checkpoint inhibitors (Sun et al., 2020). A tumor microenvironment is regulated by a variety of immunosuppressive signals. Tumor microenvironment and its heterogeneity affect patients prognosis and therapeutic response. Tumor infiltrating lymphocytes and immune scores are associated with the prognosis of pancreatic cancer and the efficacy of radiotherapy and chemotherapy. However, the underlying mechanism of immune infiltration against response in pancreatic cancer remains unclear.

As gene changes may lead to abnormal immune microenvironment in cancers, we investigated the expression of m6A-lncRNA and investigated the infiltration of immune cells in tumor tissues. In recent years, immune escape and immunosuppression have become hot spots of tumor-targeted therapy. Tumor antigen-specific T cells is a critical event for anti-tumor immune surveillance (Hegde and Chen, 2020), and macrophages play a role in the whole spectrum of tumor evolution, from initiation to metastasis (Locati et al., 2020). It is clear that M2-like macrophages can promote immunosuppression, tumor growth, and angiogenesis (Blando et al., 2019; Lin et al., 2020). TIME 2.0 was used to evaluate the immune cell infiltration.TIME2.0 utilizes an R package “immunedeconv,” which integrates six state-of-the-art algorithms, including TIMER, CIBERSORT, EPIC xCell, MCP counter, and quanTIseq (Newman et al., 2015). It shows associations between gene expression, mutations, immune infiltration, and survival features in the TCGA cohorts. In our study, we found that the proportion of naive B cell CD8^+^ T cell and Treg cells were lower in patients in the high-risk group, meanwhile M0 and M2 macrophages were higher in the high-risk group, which is similar to previous report (Blando et al., 2019). Abnormal signal transduction was also important in tumor development. T cell infiltration and its functional pathway is an evolutionarily conserved signaling pathway, which regulates tumor immunologic status, hence impact the outcome. Our new understanding of how m6A-lncRNA affects the immune microenvironment in pancreatic cancer patients may benefit future tumor targeted therapies.

There are some limitations in our study. Our findings were analyzed by bioinformatics methods and were internally validated. The accuracy of the model need to be confirmed with additional datasets. Clinical samples have been collected and we will next do validation in order to provide more evidence. The 8 m6A-lncRNA signature may provide clues for discovering the mechanisms for pancreatic cancer, but experimental study is warranted on these lncRNAs.

## Conclusion

In conclusion, our results suggested that the 8 m6A-associated lncRNAs signature could serve as a promising prognostic indicator in patients with pancreatic cancer. It will guide the development of biomarkers and targeted immune regulation in pancreatic cancer.

## Data Availability Statement

The datasets presented in this study can be found in online repositories. The names of the repository/repositories and accession number(s) can be found in the article/Supplementary Material.

## Author Contributions

SH and QY had all the data and were responsible for the decision to submit it for publication. XY was responsible for data collection and analysis and was appointed to write the draft of the manuscript. XY, PD, and YY designed and typeset all the figures and tables. FL, HW, YL, and MS were responsible for all data curation. All authors read and approved the final version of manuscript.

## Conflict of Interest

The authors declare that the research was conducted in the absence of any commercial or financial relationships that could be construed as a potential conflict of interest.

## Publisher’s Note

All claims expressed in this article are solely those of the authors and do not necessarily represent those of their affiliated organizations, or those of the publisher, the editors and the reviewers. Any product that may be evaluated in this article, or claim that may be made by its manufacturer, is not guaranteed or endorsed by the publisher.

## Footnotes

1
https://portal.gdc.cancer.gov/

2
https://gtexportal.org/home/index.html

3
http://m6a2target.canceromics.org

4
http://timer.cistrome.org
