# Supplementary material for: Identification of N6-Methyladenosine-Associated Long Non-coding RNAs for Immunotherapeutic Response and Prognosis in Patients With Pancreatic Cancer
Source: Front Cell Dev Biol. 2021 Sep 21;9:748442. doi: 10.3389/fcell.2021.748442 (PMC8490671; doi:10.3389/fcell.2021.748442)
Supplement: Supplementary file 1 [file Data_Sheet_1.doc]

**Supplementary Material**

1 SUPPLEMENTARY DATA

Pancreatic cancer samples and the corresponding clinical data were obtained from the TCGA PAAD database (http://cancergemome.nih.gov/) and normal samlple data was obtained from GTEx database(https://gtexportal.org/home/index.html ).

1.1 gtex_RSEM_gene_fpkm.gz represents data from GTEx

1.2 TCGA-PAAD.htseq_fpkm.tsv.gz represents transcription data from TCGA data base

1.3 gdc_download_20210202_043239.502504.tar.gz represents clinical data from TCGA data base

2 SUPPLEMENTARY FIGURES

Figure S1. Flowchart of this study
