# Supplementary figures and images for: Identification of N6-Methyladenosine-Associated Long Non-coding RNAs for Immunotherapeutic Response and Prognosis in Patients With Pancreatic Cancer
Source: Front Cell Dev Biol. 2021 Sep 21;9:748442. doi: 10.3389/fcell.2021.748442 (PMC8490671; doi:10.3389/fcell.2021.748442)

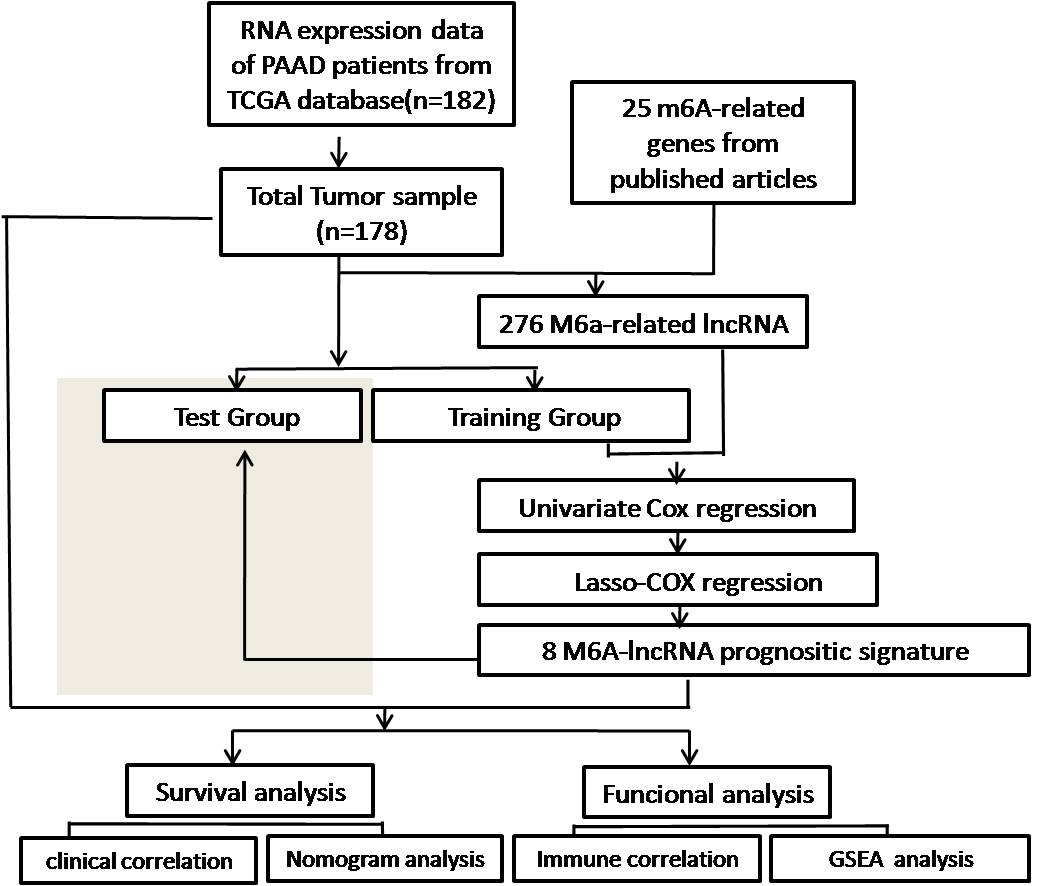

Supplement: Supplementary file 2 [file Image_1.jpg]
